# An investigation of trauma-associated appraisals and posttraumatic stress disorder in British and Asian trauma survivors: the development of the Public and Communal Self Appraisals Measure (PCSAM)

**DOI:** 10.1186/2193-1801-3-44

**Published:** 2014-01-24

**Authors:** Alberta Engelbrecht, Laura Jobson

**Affiliations:** University of East Anglia, Norwich, UK; Medical Research Council Cognition and Brain Sciences Unit, Cambridge, UK

**Keywords:** Appraisals, PTSD, Culture, Posttraumatic Cognition, PTCI, Trauma

## Abstract

Two studies examined the role of culture on cognitive appraisals of trauma and associated implications for posttraumatic psychological adjustment. Study 2 also investigated the reliability and validity of a new measure assessing public and communal aspects of trauma-associated appraisals (Public and Communal Self Appraisals Measure; PCSAM). Study 1′s non-clinical sample (*N* = 75) and Study 2′s sample of British and Asian trauma survivors with and without PTSD (*N* = 95) provided an everyday and trauma memory, completed an Appraisal Inventory, the Posttraumatic Cognitions Inventory and measures of PTSD. Study 2 participants also completed the PCSAM. Conjoined, there were cultural differences in appraisals of everyday and trauma experiences. Nonetheless, there appeared to be cultural similarities in the dysfunctional appraisals of those with PTSD. The PSCAM had good internal consistency, test-retest reliability, convergent validity, and discriminative validity. Findings are discussed in terms of combining cultural models of self with current PTSD models.

An impressive body of literature identifies several factors which impede post-trauma recovery, maintain posttraumatic symptoms and predict the development of on-going posttraumatic stress disorder (PTSD) (see Brewin et al. 2000; Ozer et al. 2003, for reviews). One such factor is negative cognitive appraisals (Kleim et al. 2007). Cognitive appraisals are of particular interest because they are central to influential clinical cognitive models of PTSD. Ehlers and Clark (2000) emphasize that self-relevant appraisals of the trauma experience and/or its sequelae function to maintain a sense of current threat in the survivor’s life and are instrumental in promoting the use of maladaptive strategies, which in turn, maintains current symptoms. Empirical evidence supports these theoretical assertions and suggests that cognitive factors are the most useful of a set of pre-trauma factors, trauma specific factors and other predictors for identifying chronic PTSD (Kleim et al. 2007). Moreover, appraisals are potentially modifiable and thus, provide important targets for treatment (Resick 2001). However, the majority of our understanding regarding the role of appraisals in PTSD is informed by research using Western populations. Despite the increase in recognition that PTSD is observed in many different societies and cultures, little is known about the etiology, maintenance and treatment of PTSD in non-Western cultures (Figueira et al. 2007; Foa et al. 2009). Accumulating research (e.g. Figueira et al. 2007; Jobson 2009) indicates PTSD to be is a universal phenomenon. While PTSD lifetime prevalence rates vary from country to country, general population studies have put this figure at between 1% and 7.8% for those from Western cultures (Kessler et al. 1995) and studies that have researched PTSD prevalence rates in non-Western countries have found comparable rates (Fu et al. 2013; Zhang et al. 2012). Given the central role of cognitive appraisals in PTSD, it is important to consider the influence of culture on the relationship between cognitive appraisals and PTSD and the use of culturally adequate and valid assessment of trauma-related appraisals in PTSD research and clinical practice (Su and Chen 2008).

The question of whether culture influences how a given everyday event is experienced has received some attention in cross-cultural psychology research where it has been found that culture influences appraisals (Mesquita and Walker 2003). One such influence on appraisals arises from cultural differences in self-construal (Markus and Kitayama 2010). In Western cultures, the self is conceived as a unique, independent, autonomous individual comprised of a particular arrangement of internal attributes (e.g., traits, abilities, motives). The goals of the independent self are to be unique, express the private self, realize internal attributes and promote personal goals. In Asian cultures, the self is perceived as an interdependent entity attending to and fitting in with others and the surrounding social context. The goals of the interdependent self are to occupy one’s proper place and engage in appropriate action (Markus and Kitayama 2010). It is important to note that while there are obviously within cultural differences, cultural differences in self-construal facilitate and foster certain appraisals of events, whilst making the incidence of other appraisals less valued and less likely (Mesquita and Walker 2003).

These theoretical propositions are supported by considerable research. Research has demonstrated Western cultures report more appraisals of perceived control, responsibility and anticipated effort than Asian cultures (e.g., Matsumoto et al. 1988; Mauro et al. 1992; Mesquita and Markus 2004; Scherer 1997) as Western cultures attach more value to agency, personal responsibility, and a personal sense of control (Fiske et al. 1998 Markus and Kitayama 1991 Nisbett et al. 2001). In contrast, personal agency and control has less applicability in Asian cultures rather interdependence of an individual and their environment is stressed (Mesquita and Walker 2003). Culture also influences how people react to different cognitive appraisals so that reactions generally correspond and reinforce cultural norms (e.g. Kim 2002; Kitayama et al. 2006; Leu et al. 2010; Mesquita and Markus 2004; Mesquita and Walker 2003). Appraisals of personal responsibility, autonomy and perceived control have been found to predict positive affect in Western cultural groups but less so for those from Asian cultures (Mesquita and Karasawa 2002; Mesquita and Walker 2003). Sato (2001) suggests that diminished levels of personal agency and perceived personal control can result in depression and anxiety in those holding a strong independent self-construal. In contrast, alienation and isolation may be more associated with depression and anxiety in those holding an interdependent self-construal.

The question remains however, how do cultural differences in self-construal influence appraisals of trauma and consequently PTSD? To date, as far as we are aware, only one study has investigated the influence of cultural differences in self-construal on trauma appraisals. Research has demonstrated that appraisals of mental defeat, control strategies, permanent change and alienation are associated with PTSD (e.g. Dunmore et al. 2001; Ehlers et al. 2000; Ehlers et al. 1998). These appraisals focus on the self and one’s actions, autonomy and consistency across time (i.e. mental defeat, control, permanent change) and on the self in relationship to others (i.e. alienation). Jobson and O’Kearney (2009) found that trauma survivors with PTSD from Western, individualistic cultures reported more mental defeat, alienation, permanent change and less control strategies than non-PTSD trauma survivors from individualistic cultures. In contrast, for those from non-Western, collectivistic cultures only alienation appraisals differentiated between trauma survivors; those with PTSD had more alienation appraisals than those without PTSD. This finding provides preliminary support for cultural differences in self influencing the relationship between appraisals and posttraumatic psychological adjustment. Namely, as Jobson (2009) argues, if appraisals of trauma challenge one’s self-construal (i.e. either the culturally emphasised independent or interdependent self) this can have an impact on PTSD risk and maintenance.

The two current studies described below aimed to extend this work. The overall objective of these studies was to investigate the influence of culture on the cognitive appraisals of trauma and associated implications for posttraumatic psychological adjustment. Specifically, the studies aimed to investigate whether similar cultural differences found in appraisals of everyday events extend to the trauma memory and to explore the relationships between these appraisals and PTSD. Secondly, research suggests that certain trauma-specific cognitive appraisals (i.e. negative cognitive appraisals about the self and world and self-blame) are associated with PTSD symptoms (e.g., Foa et al. 1999). Therefore, the two studies reported also aimed to investigate whether culture influences these trauma-specific appraisals and if so, what the consequences of these differences are for PTSD. Study 1 investigated these aims in a sample of British and Asian international university students. Study 2 extended Study 1 to a sample of British and Asian trauma survivors with and without PTSD.

## Study 1

The cross-cultural literature has, in particular, investigated the influence of culture on ten cognitive appraisal dimensions in relation to everyday events. These ten cognitive appraisals include pleasantness (result of having what one desires), attentional activity (strong motivation to attend closely to an event), certainty (predictability, certainty and understandability of the situation), coping ability (ability to cope with situation), perceived control (level of personal perceived control in the event), responsibility (personal responsibility for the event), anticipated effort (anticipate needing to expend energy or effort in the event), goal-need conduciveness (level of importance and perceived obstacles in the event), legitimacy (perceived fairness of an outcome of an event) and norm/self compatibility (appropriateness of own behaviour, feelings, thoughts and actions in the situation) (Mauro et al. 1992). However, to date, research has not investigated these cognitive appraisal dimensions in relation to the trauma memory. The trauma memory potentially differs from other autobiographical memories as, by definition, it results from an extremely stressful or traumatic event and is generally associated with an increase in emotional arousal, intensity and schema violations (Rubin et al. 2008). Moreover, as outlined above, appraisals associated with trauma are of particular relevance for those with PTSD as dysfunctional appraisals are central to the understanding of PTSD (Brewin 2011; Brewin et al. 1996; Ehlers and Clark 2000). Therefore, the first aim of Study 1 was to explore the influence of culture on these cognitive appraisal dimensions in relation to trauma and to examine the relationships between these appraisals and PTSD symptoms in British and Asian participants.

The Posttraumatic Cognitions Inventory (PTCI; Foa et al. 1999) was developed as a self-report questionnaire that is now widely used to assess negative trauma-specific cognitive appraisals. It includes three factors; negative cognitive appraisals about the self, negative cognitive appraisals about the world and self-blame. While the PTCI is widely used, as far as we are aware, only one study has investigated the reliability and validity of the PTCI for use in Asian populations. Su and Chen (2008) reported the factor structure and psychometric properties of a Chinese version of the PTCI and its relationship with PTSD symptoms. They used a sample of 240 traumatized university students in Taiwan. Their confirmatory factor analysis suggested adequate replication of the original three-factor structure of the PTCI after eliminating four cross-loaded items. Their 29-item PTCI was found to have good psychometric properties and had moderate to high correlations with PTSD symptoms. This initial study suggests that similar negative cognitions may contribute to PTSD development in Asian samples. The four items on the PTCI that were excluded on the Chinese version of this instrument were 1) “My reactions since the event mean that I am going crazy” 2) “I feel isolated and set apart from others” 3) There is something wrong with me as a person” 4) “Somebody else would not have gotten into this situation”. The first three items are from the negative self-subscale and the last item is from the self-blame subscale. As Su and Chen (2008) assert, these items were excluded because of cross-loading across different factors, while the last item did not load on the BLAME factor but rather the SELF factor. This could be due to cultural deviations regarding self-blame attributions and the manner in which the self is evaluated. For instance, in regards to self-blame, Su and Chen (2008) highlight that Chinese trauma victims may still consider the possibility of others encountering a similar situation, while it could also be inferred that Chinese victims may assign less responsibility to themselves in causing trauma than Western victims do. Consequently, in both instances the Chinese participants may attribute less self-blame to the trauma experience. The exclusion of these items, however, highlights cultural concerns and potentially point to the PTCI being less appropriate for assessment of posttraumatic negative cognitions in Asian populations. The second aim of Study 1, therefore, is to further investigate the influence of culture on trauma-specific negative cognitive appraisals by examining whether there are cultural differences in these trauma-specific dysfunctional cognitive appraisals and the relationships between these trauma-specific cognitive appraisals and PTSD symptoms.

## Method

### Participants

Participants were white British students (*n* = 34) and Asian international students (*n* = 41; Chinese *n* =28, East-Asian *n* = 13) who were recruited from undergraduate and postgraduate courses at the University of East Anglia. Participants received £5 for their participation. All participants had to be able to complete the questionnaire booklet in English.

### Measures

#### Appraisal variables

Participants were asked to recall a positive and a trauma memory. The trauma event was subjectively selected by the participant as the most traumatic event in their life and thus, not all events fulfilled PTSD criterion A (American Psychiatric Association 2000). To encourage participants to think deeply about each memory, they were asked to describe the event in detail.

#### Appraisal inventory (AI)

Following each memory, participants completed the AI. The AI was originally developed by Mauro et al. (1992) to investigate cross-cultural differences in appraisals. It consists of 28 questions around ten appraisal dimensions (pleasantness, attentional activity, certainty, coping ability, perceived control, responsibility, anticipated effort, goal/need conduciveness, legitimacy and norm/self-compatibility) related to specified events. Participants scored responses in relation to the positive and trauma memory on seven-point scales from 1 (not at all) to 7 (very much).

#### PTCI (Foa et al. 1999)

The PTCI is a 33-item inventory assessing appraisals related to trauma. Items are rated on seven-point scales. The PTCI is a well-established inventory (Beck et al. 2004; Foa et al. 1999; Van Emmerik et al. 2006) and has been used cross-culturally (Dragan et al. 2005 Su and Chen 2008). The PTCI has three subscales; appraisals about negative self, negative world and perceived self-blame regarding the trauma. In the current study the total scale and subscales demonstrated good internal consistency; PTCI total α = .75; Negative self α = .76; Negative world α = .80; Self-blame α = .79.

#### Psychological adjustment

Posttraumatic stress symptoms were assessed using the widely used self-report questionnaire, Impact of Event Scale-Revised (IES-R; Weiss and Marmar 1997). The IES-R is a standard measure used to assess PTSD symptomatology. It consists of three subscales for avoidance, intrusions and hyperarousal symptoms relating to a specific event; in this instance it was the trauma memory disclosed. It has been used in previous cross-cultural research (e.g. Jobson and O’Kearney 2006). In the current study the IES-R demonstrated good internal consistency (Cronbach’s α = .87).

Depression was measured using Part II of the Hopkins Symptom Checklist (HSCL-25) which has 15 items that assess depression symptoms (Derogatis et al. 1974). It has good psychometric properties and is regularly used in cross-cultural research (e.g., Jobson and O’Kearney 2006). In the current study the depression subscale demonstrated good internal consistency (Cronbach’s α = .76).

### Procedure

Ethical approval was obtained from University of East Anglia (reference number 2009/10-029). Participants met with the researcher and completed the questionnaires in the following order: trauma memory and appraisals, positive memory and appraisals (the positive and trauma memories were counterbalanced), IES-R, PTCI and HSCL-25. Participants also provided information on their age, gender, length of time in the UK, cultural identity and rated, on a 10-point scale from 1 (*not at all*) to 10 (*extremely*), how difficult they had found the study.

## Results

### Participant characteristics

Participant characteristics and group comparisons are presented in Table 1. The British group had, unsurprisingly, lived in the United Kingdom for a significantly longer period of time and reported a significantly higher level of English ability than the Asian group. The Asian group had significantly higher levels of PTSD symptoms and significantly less depression symptoms than the British group. The groups did not differ significantly in terms of type of trauma or positive event disclosed.

**Table 1:** **Mean and (standard deviations) for participant characteristics and group comparisons**

	British	Asian	***t***	***p***
Age (years)	23.00 (6.27)	23.02 (4.18)	.20	.98
Time in UK (years)	20.56 (6.71)	1.39 (2.00)	16.08	< .001
Self-rated English ability	9.06 (1.15)	5.78 (1.90)	9.18	< .001
Task difficulty	4.35 (2.19)	5.12 (1.85)	1.65	.10
IES-R	16.65 (17.33)	30.44 (15.50)	.36	.001
HSCL	1.90 (.58)	1.60 (.47)	2.47	.02
Years since trauma	7.88 (6.91)	6.49 (6.57)	.86	.39
Years since pleasant event	3.45 (3.99)	3.63 (4.73)	.16	.88
Trauma type (*n*)	Death/illness = 12; Accident = 3; Assault = 7; Life stressor = 12	Death/illness = 9; Accident = 9; Assault = 5; Life stressor = 18	-	-
Positive type (*n*)	Achievement = 20; Relationship = 12	Achievement = 28; Relationship = 7	-	-

*Note*: IES-R = Impact of Event Scale-Revised. HSCL = Hopkins Symptom Checklist. Life Stressor included academic stress, relationship stress or stress associated with moving.

### Differences in magnitude of appraisals

Table 2 shows the means for each of the appraisals for both British and Asian groups. 2 (between subject; culture: British vs. Asian) x 2 (within subject; memory: positive vs. trauma) mixed analysis of variance (ANOVAs) were used with each appraisal type as the dependent variable. The findings were similar when the IES-R and depression were included as a covariate suggesting that group differences in level of posttraumatic distress did not influence the findings.

**Table 2:** **Mean and (standard deviation) for the Asian and British Group on appraisals for pleasant and trauma experiences**

	Asian		British	
	Pleasant	Trauma	Pleasant	Trauma
Pleasantness	5.85 (2.19)	4.41 (2.11)	4.91 (2.57)	3.35 (2.55)
Coping ability	5.88 (1.91)	4.39 (2.41)	6.29 (2.25)	3.91 (2.27)
Anticipated effort	11.85 (3.94)	13.49 (2.68)	12.85 (4.47)	15.26 (2.29)
Legitimacy	15.00 (2.53)	8.66 (4.37)	15.21 (4.04)	5.94 (4.01)
Norm/Self	12.49 (2.96)	11.34 (3.66)	16.65 (1.79)	14.21 (3.37)
Goal/Need	19.27 (3.79)	19.44 (4.59)	19.29 (4.83)	19.94 (4.74)
Attentional activity	25.41 (4.14)	20.95 (4.25)	28.09 (4.82)	22.59 (6.54)
Certainty	27.44 (7.68)	23.39 (7.77)	25.94 (10.07)	22.29 (10.26)
Responsibility	22.32 (3.91)	21.39 (4.95)	26.21 (5.59)	21.85 (5.89)
Perceived control	21.71 (3.95)	19.32 (4.29)	23.94 (5.92)	18.62 (6.40)
PTCI-Total	-	9.19 (1.73)	-	8.07 (1.82)
PTCI-Self	-	6.66 (1.65)	-	5.84 (1.39)
PTCI-World	-	4.89 (1.15)	-	4.22 (1.30)
PTCI-Self blame	-	3.80 (.81)	-	3.33 (1.10)

Note: PTCI = Posttraumatic Cognitions Inventory.

#### Pleasantness

There was a significant memory main effect, *F*(1, 73) = 17.26, *p* < .001, ƞ_p_^2^ = .19; unsurprisingly, the pleasant memory was appraised as being significantly more pleasant than the trauma memory. There was also a significant culture main effect, *F*(1, 73) = 6.07, *p* = .02, ƞ_p_^2^ = .08; the Asian group appraised the memories as being significantly more pleasant than the British group. The interaction was not significant.

#### Coping ability

There was a significant memory main effect, *F*(1, 73) = 33.30, *p* < .001, ƞ_p_^2^ = .31; participants appraised that they had significantly less ability to cope in the trauma memory than the pleasant memory. The culture main effect and interaction were not significant.

#### Anticipated effort

There was a significant memory main effect, *F*(1, 73) = 14.89, *p* < .001, ƞ_p_^2^ = .17; participants rated that they significantly anticipated greater need to expend energy or effort in the trauma event than the pleasant event. The culture main effect was also significant, *F*(1, 73) = 5.26, *p* = .03, ƞ_p_^2^ = .07; the British group reported significantly greater anticipated effort appraisals than the Asian group. The interaction was not significant.

#### Legitimacy

The interaction was significant, *F*(1, 73) = 5.07, *p* = .03, ƞ_p_^2^ = .07. Post-hoc comparisons revealed that while the cultural groups did not differ in terms of legitimacy of the pleasant memory, the Asian group reported the trauma memory was significantly more legitimate than the British group, *t*(73) = 2.78, *p* < .01, *d* = .65.

#### Norm/self compatibility

There was a significant memory main effect, *F*(1, 73) = 17.83, *p* < .001, ƞ_p_^2^ = .20; participants’ appraised their own behaviour, feelings, thoughts and actions to be significantly less appropriate in the trauma memory than the pleasant memory. The culture main effect was also significant, *F*(1, 73) = 38.07, *p* < .001, ƞ_p_^2^ = .34; the British group had significantly higher levels of norm-self compatibility appraisals than the Asian group. The interaction was not significant.

#### Goal/Need conduciveness

The main effects and interaction were not significant.

#### Attentional activity

There was a significant memory main effect, *F*(1, 73) = 39.89, *p* < .001, ƞ_p_^2^ = .35; participants reported significantly less motivation to attend closely during the trauma event that the pleasant event. There was a significant culture main effect, *F*(1, 73) = 6.64, *p* = .01, ƞ_p_^2^ = .08; the British group had significantly higher levels of attentional activity appraisals than the Asian group. The interaction was not significant.

#### Certainty

There was a significant memory main effect, *F*(1, 73) = 14.82, *p* < .001, ƞ_p_^2^ = .17; participants rated that the pleasant memory was significantly more predictable, certain and understandable than the trauma memory. The culture main effect and interaction were not significant.

#### Responsibility

The interaction was significant for appraisals of responsibility, *F*(1, 73) = 5.80, *p* = .02, ƞ_p_^2^ = .07. Post-hoc follow-up analyses revealed that the British group reported that they were significantly more personally responsible for the pleasant memory than the Asian group, *t*(73) = 3.54, *p* = .001, *d* = .80. However, the cultural groups did not differ significantly in terms of personal responsibility for the trauma memory and the British group reported significantly lower levels of personal responsibility in the trauma memory when compared to the pleasant memory, *t*(33) = 3.86, *p* < .001, *d* = .76.

#### Perceived control

There was a significant memory main effect, *F*(1, 73) = 24.56, *p* < .001, ƞ_p_^2^ = .25; participants reported that they had significantly greater perceived control in the pleasant memory than the trauma memory. The culture main effect and interaction were not significant.

### PTCI

To compare British and Asian participants’ responses on the PTCI, a multivariate analysis of variance (MANOVA) was carried out with the total PTCI and three subscales as dependent variables. The multivariate effect of Group was not significant, Wilks’ Lambda = .92, *F*(3, 71) = 2.16, *ns*, ƞ_p_^2^ = .08.

### Associations between trauma appraisals and PTSD symptoms

Given the IES-R was not normally distributed and transformations did not achieve normality, Spearman correlations were used. Table 3 shows a significant negative correlation between perceived personal control and PTSD symptoms for the British group. The Asian group had a significant correlation between attentional activity and PTSD symptoms. None of the correlation coefficients differed significantly between the two groups.

**Table 3:** **Spearman correlation coefficients (two-tailed) between trauma appraisals and PTSD symptoms for the British and Asian Cultural Groups**

	British	Asian
Pleasantness	.03	-.22
Attentional activity	-.33	-.50**
Certainty	.14	-.05
Coping ability	-.21	-.13
Perceived control	-.35*	-.17
Responsibility	-.10	-.17
Anticipated effort	.04	.13
Goal/Need	-.01	.12
Legitimacy	-.16	-.09
Norm/Self	.18	.12
PTCI total	.40*	.29
PTCI self	.49**	.38*
PTCI world	.39*	.07
PTCI self-blame	.15	.01

Note: *p < .05. **p < .01*.*PTCI = Posttraumatic Cognitions Inventory.

Table 3 also shows that for the British group, PTSD symptoms and the PTCI were significantly correlated. Additionally, the PTCI significantly predicted PTSD symptoms, *R*^*2*^ = .18, *β* = .42, *SE* = .09, *t* = 2.65, *p* = .01. In contrast, for the Asian group, even though this group had higher levels of PTSD symptoms, only PTCI negative self subscale was significantly correlated with PTSD symptoms and the PTCI did not significantly predict PTSD symptoms, *R*^*2*^ = .06, *β* = .25, *SE* = .14, *t* = 1.62, *p* = .11. This result suggests that the PTCI may better account for PTSD symptoms in the British group than the Asian group. We also explored these correlations for the Asian group excluding the items that were excluded from the Chinese version of the PTCI (Su and Chen 2008). When this was the case, the PTCI was now significantly correlated with, *r*(41) = .31, *p* < .05, and predicted, *R*^*2*^ = .10, *β* = .17, *SE* = .08, *t* = 2.09, *p* = .04, PTSD symptoms.

## Discussion

Study 1 found that pleasant and trauma memories are appraised differently. Despite this, the British group, regardless of memory type, reported higher levels of anticipated effort, attentional activity and norm-self compatibility appraisals and lower levels of pleasantness appraisals than Asian participants. This aligns with previous cross-cultural research and reflects British participants valuing agency, assuming their reactions are typical and being less concerned about discrepancies with the reactions of others (Markus and Kitayama 2010; Mesquita and Walker 2003). The trauma memory was only unique in terms of legitimacy and responsibility appraisals. The Asian group reported the trauma memory was more legitimate than the British group. This may reflect Asian cultures having greater acceptance of situation outcomes and fate (Mesquita and Walker 2003). The British group, as in previous research, had significantly higher levels of appraisals of responsibility than the Asian group for the pleasant memory. However, for the trauma memory the British group did not differ from the Asian group and had reduced their appraisals of responsibility to a level equivalent to the Asian group. Given the importance of responsibility in Western cultures, participants from British cultures may not want to feel responsible for trauma events, which may challenge and threaten the independent self.

Finally, a significant negative correlation was found between lower levels of perceived control and PTSD symptoms for the British group. Appraisals of control are valued in Western cultures and the violation of expectations/cultural norms in appraisals can lead to distress (Mesquita and Walker 2003). Therefore, for British participants less perceived control may be associated with posttraumatic distress. While for the British group, PTCI appraisals were significantly correlated with, and predicted, PTSD symptoms, for the Asian group, the PTCI did not significantly predict PTSD symptoms. This result suggests that the PTCI may better account for PTSD symptoms in the British group than the Asian group. One possibility for this may be the PTCI assesses individualistic-type appraisals (e.g. I am a weak person, I have permanently changed for the worse, I can’t rely on myself, I am inadequate) rather than interdependent, public (i.e. social roles and identities) and communal (relationships and interdependence) appraisals, which are emphasised in Asian cultures. However, when only the items on the Chinese version of the PTCI (Su and Chen 2008) were used, the PTCI did significantly correlate with and predict PTSD symptoms. This suggests that the 29-item PTCI may be more appropriate in Asian samples.

The generalizability of these findings is potentially limited by the fact that participants were university students and not all participants were trauma survivors. Furthermore, Study 1 would be strengthened by focusing on those with and without a diagnosis of PTSD. Nevertheless, Study 1 found cultural differences in the appraisals of everyday events and trauma experiences. The findings also suggested that culture may influence the relationships between trauma–related appraisals and PTSD symptoms. Thus, further research in this area was deemed to be warranted.

## Study 2

The aim of Study 2 was to further investigate the influence of culture on trauma-related appraisals using British and Asian trauma survivors with and without PTSD. Study 1’s findings suggested that culture may influence the relationships between trauma–specific appraisals and PTSD symptoms. It was found that the PTCI was associated with PTSD symptoms more strongly in the British group than in the Asian group and while the PTCI significantly predicted PTSD symptoms in the British group, for the Asian group the PTCI did not significantly predict PTSD symptoms. Thus, the PTCI may not fully assess trauma-specific appraisals associated with PTSD in those from Asian cultures. We suggest that this may be the result of the PTCI typically tapping into individualistic-type appraisals rather than more interdependent, public and communal appraisals.

Therefore, following Study 1 we conducted a qualitative study exploring cognitive appraisals that were associated with trauma and disrupted psychological adjustment following trauma in Asian trauma survivors. Key informant interviews with mental health practitioners who work with trauma survivors from Asian cultures and three focus groups comprised of trauma survivors from Asian cultures were selected to generate a greater understanding of culturally-appropriate appraisals. We used open-ended interviews to collect data. We also asked participants to rate the appropriateness of the PTCI items for use in Asian cultures. Using template analysis several strong emergent themes were elicited that focused on a) social and cultural roles following a trauma, b) relationships to others following trauma, and c) appraisals of one’s belief systems following the traumatic incident^a^. These themes seemed to align with cross-cultural research on self-construal (i.e. public and communal aspects of self) and the influence of these differences on appraisal tendencies. Further, the beliefs theme reflects cross-cultural research on self-control and external attribution of failure (see Ji et al. 2000; Sastry and Ross 1998; Tweed et al. 2004).

The findings of this qualitative research were used to develop a new measure; the Public and Communal Self Appraisal Measure (PCSAM). The items on the PCSAM were developed to represent potential dysfunctional cognitions as a result of a) trauma leading to disintegration in one’s cultural/social roles, b) dysfunctional appraisals about communal aspects of self and relationships, and c) disintegration in one’s belief system.

The aim of Study 2 was therefore to investigate the a) aims of Study 1 using British and Asian trauma survivors with and without PTSD and, b) reliability and validity of the PCSAM and its appropriateness for use in Asian trauma survivor populations.

## Method

### Design

The study adopted a cross-sectional 2 (culture: British vs. Asian) x 2 (memory: trauma vs. negative) x 2 (PTSD condition: PTSD vs. no-PTSD) design with the first and third variables being between group and the second variable being within group. The study also piloted the PCSAM to investigate whether it is a valid and reliable measure of dysfunctional appraisals associated with PTSD. To assess reliability of the PCSAM, the PCSAM was also administered two weeks later (Time 2).

### Participants

All participants (*N* = 95) were recruited using from the general community in the UK by posters in public places, Adult Migrant English Programs, advertisements in local and ethnic newspapers, contacts with ethnic organizations and communities and organizations that provide treatment for trauma survivors. Notices called for those who had experienced a traumatic event and identified the study as researching trauma, appraisals and culture. The Asian group was comprised of Chinese (*n* = 12), Japanese (*n* = 18), Korean (*n* = 2), and South Asian (*n* = 15) participants.

### Measures

#### PCSAM

The items for the PCSAM were developed from the findings of the qualitative study. The original PCSAM consisted of 21 items that were thought to relate to three sub-scales; 1/ potential dysfunctional cognitions as a result of world/external causes (1. Fate/God/bad luck caused the event to happen; 2. Since the event I have a pessimistic view of life; 3. My faith/religion/beliefs have been challenged by the event; 4. Since the event I feel let down by the world; 5. Since the event I feel let down by fate/my beliefs/God/my faith; 6. Since the event I do not feel like I have a place in the world), 2/ communal (7. Since the event I have sacrificed my needs for the needs of significant others; 8. Since the event I feel like I am a burden to others; 9. I do not want anyone to know about the event; 10. Since the event I no longer feel close to others; 11. Since the event other people have become a priority; 12. Since the event my relationships have been damaged or challenged; 13. Since the event I find it hard to have relationships with others; 14. Since the event others have made the problem worse), and 3/ disintegration from cultural/social roles (15. Since the event I have lost my social role/identity (e.g. as a parent, husband, wife, at work); 16. Since the event I have failed in my role(s); 17. Since the event my values have changed; 18. Since the event I try harder to meet social or cultural expectations; 19. Since the event I have not lived up to social or cultural expectations; 20. Since the event I try to act appropriately; 21. Since the event I do not feel I am a significant member of my culture/society/community/group). Participants were asked to rate these items in relation to the trauma disclosed on the Structured Clinical Interview for DSM-IV-TR Axis I Disorders (SCID-I; First et al. 2002). Participants were instructed ‘Please read each item and then indicate how much you agree with each statement in regards to the past seven days’. Items were rated on Likert-type rating scales ranging from 1(*totally disagree*) to 7 (*totally agree*).

#### PTSD checklist (PCL; Weathers et al. 1993)

The PCL is a 17-item self-report measure of the DSM-IV symptoms of PTSD (American Psychiatric Association 2000). The PCL is used to screen individuals for PTSD, diagnosing PTSD and monitoring symptom change during and after treatment. Of the three versions of the PCL, the PCL-C (civilian) was used and asked about symptoms in relation to the traumatic experience the participant’s referred to in Task 1 of the Structured Clinical Interview for DSM-IV-TR Axis I Disorders (SCID-I; First et al. 2002). Since its introduction the PCL-C has been widely used in research and clinical settings. The PCL is scored as a total symptom severity score (range = 17–85) and has good psychometric properties (Keen et al. 2008).

### Procedure

Ethical approval was obtained from NRES Committee East of England, Essex REC (reference number 12/EE/0194. PTSD diagnosis was identified using the Overview and PTSD module from the SCID-I for DSM-IV-TR Axis I Disorders (First et al. 2002). The SCID-I is a semi-structured interview and is routinely used as a diagnostic instrument. Interviews were audio-recorded to account for inter-rater agreement and reliability of the coding of the data. Inter-rater reliability was found to be good (Kappa coefficient of .88) and discrepancies were resolved between raters. Participants were then asked to provide their trauma memory and a negative memory (counterbalanced). In Study 2 a negative memory was selected as a comparison memory given it should be closer in valence to a trauma memory than a positive memory (Study 1). The rationale for this choice was based on understandings that differences in appraisals between the two would highlight acute differences between the two types of memories. Thus using another form of unwanted memories (e.g. negative memories) is helpful in understanding how people with traumatic memories, such as those with PTSD will cope and appraise these particular, unique events. Following each of these memories participants completed the AI as described in Study 1. The HSCL and PTCI (used in Study 1) and then the PCL and PCSAM were administered. Two weeks later the PCSAM was re-administered to examine test-retest reliability. As in Study 1, participants provided information on their age, gender, length of time in the UK, cultural identity and task difficulty.

## Results

### Participant characteristics

Participant characteristics are presented in Table 4. The cultural groups did not differ significantly in terms of education or task difficulty but did differ significantly in age *F*(1, 91) = 9.71, *p* < .01, ƞ_p_^2^ = .10, gender; χ^2^ (1, *N* = 95) = 4.88, *p* < .05, length of time in the UK, *F*(1, 91) = 145.07*, p* < .001, ƞ_p_^2^ = .61, and self-rated English ability, *F*(1, 91) = 34.10, *p* < .001, ƞ_p_^2^ < .01. As expected, those with PTSD scored significantly higher on the PCL than those without PTSD, *F*(1, 91) = 154.17, *p* < .001, ƞ_p_^2^ = .63. The cultural main effect and interaction were not significant. Those with PTSD also had significantly higher symptoms of depression than those without PTSD, *F*(1, 91) = 59.52, *p* < .001, ƞ_p_^2^ = .40. The culture main effect and interaction were not significant. There was a group difference in trauma type χ^2^ (4, *N* = 95) = 10.36, *p* = .04. There were no PTSD group differences in trauma type.

**Table 4:** **Means and (standard deviations) for participant characteristics**

	British		Asian	
	PTSD	No PTSD	PTSD	No PTSD
	(***n*** = 15)	(***n*** = 33)	(***n*** = 19)	(***n*** = 28)
Gender (*n*)	Male = 7	Male = 18	Male = 4	Male = 10
Age (*in years*)	41.60 (12.40)	34.21 (8.30)	33.11 (10.06)	28.21 (8.83)
Years spent in UK	40.93 (12.44)	30.17 (8.45)	7.13 (10.99)	7.31 (10.01)
Task difficulty	5.80 (2.17)	4.39 (2.21)	4.45 (1.72)	4.57 (2.39)
English ability	9.00 (1.00)	8.58 (1.37)	6.47 (1.90)	6.96 (1.93)
Education (*n*)	Secondary = 7, Degree = 6, Postgrad = 2	Secondary = 10, Degree = 14, Postgrad = 9	Secondary = 10, Degree = 2, Postgrad = 7	Secondary = 7, Degree = 8, Postgrad = 13
PCL	42.47 (6.99)	22.50 (4.85)	47.70 (12.35)	23.89 (8.23)
HSCL	35.33 (8.25)	20.73 (4.98)	32.74 (9.24)	23.45 (7.69)
Trauma type (*n*)	Accident = 6	Accident = 18	Accident = 6	Accident = 13
	Disaster = 1	Disaster = 3	Disaster = 6	Disaster = 6
	Combat = 2	Combat = 6	Assault = 5	Combat = 1
	Assault = 4	Assault = 5	Death = 2	Assault = 6
	Death = 1	Death = 1		Death = 2

Note: Secondary = Completed secondary school. Postgrad = Completed postgraduate degree. PCL = Post-traumatic Stress Disorder Checklist. HSCL = Hopkins Symptom Checklist. Disaster = Natural Disaster. Assault includes sexual and non-sexual. Death = witness sudden death.

### Appraisals

Table 5 shows the means for the appraisal measures. 2 (Culture: Asian vs. British) x 2 (PTSD condition: PTSD vs. non-PTSD) x 2 (Memory: Negative vs. Trauma) mixed ANOVAs were used with each appraisal type as the dependent variable.

**Table 5:** **Mean and (Standard Deviation) for the British and Asian trauma survivors with and without PTSD on appraisals associated with negative and trauma memories**

	British		Asian	
	PTSD	No PTSD	PTSD	No PTSD
Pleasantness				
Negative	2.73 (1.94)	4.03 (1.85)	4.15 (2.52)	4.82 (2.54)
Trauma	2.07 (1.39)	3.81 (2.34)	2.84 (2.48)	4.39 (2.23)
Coping ability				
Negative	4.67 (2.50)	6.21 (2.00)	5.53 (2.39)	5.07 (1.56)
Trauma	2.80 (1.86)	5.33 (2.82)	2.79 (1.65)	5.04 (2.38)
Anticipated effort				
Negative	13.20 (3.55)	12.79 (3.14)	13.11 (3.77)	12.25 (3.22)
Trauma	15.73 (2.15)	14.85 (3.24)	15.11 (2.71)	12.14 (4.34)
Legitimacy				
Negative	7.27 (5.13)	6.39 (3.29)	7.47 (4.29)	8.82 (4.76)
Trauma	5.07 (3.90)	4.97 (3.62)	5.16 (3.64)	6.79 (4.32)
Norm/Self				
Negative	12.80 (4.13)	14.18 (2.97)	13.32 (3.81)	12.82 (3.27)
Trauma	13.26 (3.39)	14.70 (4.10)	12.74 (4.33)	13.61 (2.99)
Goal/Need				
Negative	21.00 (4.74)	20.61 (3.71)	19.47 (4.69)	18.82 (4.41)
Trauma	21.60 (3.70)	21.82 (4.23)	19.37 (5.84)	18.64 (5.13)
Attentional activity				
Negative	24.07 (4.56)	23.06 (3.68)	23.00 (5.40)	23.54 (5.36)
Trauma	24.67 (4.10)	25.52 (5.11)	20.05 (5.67)	24.29 (5.28)
Certainty				
Negative	25.67 (6.68)	23.91 (8.38)	23.95 (7.58)	23.00 (7.99)
Trauma	16.47 (9.19)	25.30 (7.54)	17.21 (8.59)	20.68 (8.59)
Responsibility				
Negative	20.47 (5.50)	24.09 (6.16)	19.95 (7.14)	21.32 (8.18)
Trauma	19.53 (7.41)	20.21 (7.61)	20.79 (5.69)	18.36 (6.86)
Perceived control				
Negative	21.73 (6.15)	23.33 (6.36)	19.26 (7.58)	20.14 (6.55)
Trauma	15.60 (6.94)	19.48 (5.38)	19.58 (6.53)	16.00 (7.78)
PTCI-Total	109.00 (40.82)	59.82 (22.91)	129.15 (33.54)	73.04 (28.46)
PTCI-Self	64.47 (25.22)	30.27 (10.80)	76.10 (23.47)	37.78 (18.09)
PTCI-World	30.13 (13.01)	20.52 (12.28)	35.55 (10.37)	24.56 (11.65)
	14.40 (7.43)	9.03 (4.93)	17.50 (6.97)	10.70 (6.47)
PTCI-Self blame				
PCSAM-Total	50.00 (17.23)	24.79 (10.34)	50.95 (10.05)	29.68 (11.98)
PCSAM-Public	16.87 (6.44)	5.64 (2.93)	14.32 (5.87)	7.64 (4.77)
PCSAM-Communal	22.13 (6.52)	12.64 (6.73)	21.58 (4.14	14.26 (7.45)
PCSAM-Beliefs	11.00 (6.81)	6.52 (3.32)	15.05 (5.95)	7.75 (3.91)

*Note:* PTCI = Posttraumatic Cognitions Inventory, PCSAM = Public and Communal Self Appraisal Measure.

#### Pleasantness

The memory main effect was significant, *F*(1, 91) = 6.24, *p* = .01, ƞ_p_^2^ = .06. As in Study 1, the traumatic memory was rated as being less pleasant than the negative memory. The cultural main effect was significant, *F*(1, 91) = 4.98, *p* = .03, ƞ_p_^2^ = .05. As in Study 1, the Asian group rated the memories to be more pleasant than the British group. The PTSD main effect was also significant, *F*(1, 91) = 10.85, *p* = .001, ƞ_p_^2^ = .11; the PTSD group found the memories to be less pleasant than the non-PTSD group. None of the interactions were significant.

#### Coping ability

Only the memory x PTSD interaction was significant, *F*(1, 91) = 9.36, *p* < .01, ƞ_p_^2^ = .93. Post-hoc comparisons revealed no difference between the groups for appraisals associated with the negative memory. However, in terms of the trauma memory those without PTSD had significantly higher appraisals of coping than those with PTSD, *t*(93) = 4.81, *p* < .001, *d* = 1.00. Paired comparisons found that while those without PTSD reported similar levels of coping appraisals in both the trauma and negative memories, those with PTSD reported lower levels of coping appraisals in the trauma memory compared to the negative memory, *t*(33) = 4.65, *p* < .001, *d* = 1.62.

#### Anticipated effort

There was a significant memory main effect, *F*(1, 91) = 12.60, *p* = .001, ƞ_p_^2^ = .12. As in Study 1, greater anticipated effort was appraised in the trauma memory than the negative memory. The culture main effort was approaching significance, *F*(1, 91) = 3.09, *p* = .08, ƞ_p_^2^ = .03. The direction of the culture main effect reflected cross-cultural and Study 1′s findings, with British participants reporting greater appraisals of anticipated effort than their Asian counterparts. There was a PTSD main effect, *F*(1,91) = 5.14, *p* = .03, ƞ_p_^2^ = .05; the PTSD group reported greater appraisals of anticipated effort than the no PTSD group. None of the interactions were significant.

#### Legitimacy

There was a significant memory main effect, *F*(1,91) = 11.72, *p* = .001, ƞ_p_^2^ = .114; participants felt the negative memory to be fairer than the trauma memory. There was also a culture main effect, *F*(1, 91) = 8.11, *p* < .01, ƞ_p_^2^ = .08; Asian participants perceived the memories to be fairer than the British group. There was no PTSD main effect and none of the interactions were significant.

#### Norm/Self compatibility

None of the main effects or interactions were significant.

#### Goal/Need conduciveness

There was only a significant culture main effect, *F*(1, 91) = 7.00, *p* = .01, ƞ_p_^2^ < .01; the British group had significantly greater goal/need conduciveness than their Asian counterparts.

#### Attentional activity

The memory x PTSD interaction was significant, *F*(1, 91) = 4.70, *p* = .03, ƞ_p_^2^ = .05. Post-hoc follow-up comparisons revealed those with PTSD reported significantly less attentional activity appraisals in the trauma memory than those without PTSD, *t*(93) = 2.53, *p* = .01, *d* = .52. However, for the negative memory there was no significant difference between groups. Paired comparisons found that those without PTSD had significantly greater appraisals of attentional activity in the trauma memory than the negative memory, *t*(60) = 2.35, *p* = .02, *d* = .61. However for those with PTSD there was no significant difference between the memories. The memory x culture interaction was also significant, *F*(1, 91) = 4.20, *p* = .04, ƞ_p_^2^ = .04. Post-hoc comparisons revealed that while the British group had significantly greater attentional activity for trauma memory than their Asian counterparts, *t*(93) = 2.46, *p* = .02, *d* = .51, no such differences were found for the negative memory. Paired comparisons found that the British group had significantly greater appraisals of attentional activity in the trauma memory when compared to the negative memory, *t*(47) = 2.61, *p* = .01, *d* = .76. However Asian participants did not significantly differ between memories. The PTSD x culture and three-way interactions were not significant.

#### Certainty

Only the memory x PTSD interaction was significant, *F*(1, 91) = 12.08, *p* = .001, ƞ_p_^2^ = .12. Post-hoc comparisons found that those with PTSD reported less certainty in the trauma memory than the no PTSD participants, *t*(93) = 3.48, *p* = .001, *d* = .72. However, the groups did not differ significantly in terms of the negative memory. Paired comparisons found that for those with PTSD there was significantly lower levels of certainty appraisals in the trauma than the negative memory, *t*(33) = 3.80, *p* < .001, *d* = 1.32. However for those without PTSD there was no significant difference between the trauma and negative memories.

#### Responsibility

Only the memory x PTSD interaction was approaching significance, *F*(1, 91) = 3.63, *p* = .06, ƞ_p_^2^ = .04. Post-hoc follow-up paired-comparisons revealed those without PTSD reported significantly greater personal responsibility for the negative event than trauma event, *t*(60) = -3.14, *p* < .01, *d* = .81. However, those with PTSD reported similar levels of responsibility appraisals in both the trauma and negative memory.

#### Control

There was a significant three-way interaction, *F*(1, 91) = 4.17, *p* = .04, ƞ_p_^2^ = .04. Post-hoc comparisons revealed that appraisals of control did not differ for Asian trauma survivors with and without PTSD for the negative or trauma memories. For British trauma survivors, while appraisals of control did not differ between those with and without PTSD for the negative memory, for the trauma memory British trauma survivors with PTSD had lower levels of control appraisals in the trauma memory than those without PTSD, *t*(46) = 2.12, *p* = .04, *d* = .63.

### Trauma specific appraisals

A 2 (Culture; Asian vs. British) x 2 (PTSD status; PTSD vs. no PTSD) ANOVA was used with PTCI total as the dependent variable. The PTSD main effect was significant, *F*(1, 91) = 66.42, *p* < .001, ƞ_p_^2^ = .42; those with PTSD scored higher on the PTCI than those without PTSD. The culture main effect was also significant, *F*(1, 91) = 6.67, *p* < .01, ƞ_p_^2^ = .07; the Asian group scored significantly higher than the British group. Contrary to Study 1, the interaction was not significant (*F* < 1); suggesting that the PTCI differentiated between those with and without PTSD regardless of trauma survivors’ cultural background.

A 2 (Culture; Asian vs. British) x 2 (PTSD status; PTSD vs. no PTSD) MANOVA was used with PTCI sub-scales as the dependent variables. The multivariate effect of Group was not significant, Wilks’ Lambda = .95, *F*(3, 89) = 1.69, *ns*, ƞ_p_^2^ = .05. The multivariate effect of PTSD was significant, Wilks’ Lambda = .53, *F*(3, 89) = 26.06, *p* < .001, ƞ_p_^2^ = .46. Follow-up analyses found that the PTSD group scored significantly higher on all subscales than those without PTSD (negative self, *F*(1, 91) = 74.04, *p* < .001, ƞ_p_^2^ = .45, negative world, *F*(1, 91) = 14.40, *p* < .001, ƞ_p_^2^ = .14, self-blame, *F*(1, 91) = 21.56, *p* < .001, ƞ_p_^2^ = .19). The multivariate effect of the interaction was not significant, Wilks’ Lambda = .99, *F*(3, 89) = .28, *ns*, _p_^2^ = .009. Additionally, unlike in Study 1, the PTCI correlated significantly with PTSD symptoms (PCL) in both cultural groups; Asian PTCI, *r*(47) = .72, *p* < .001, Negative self, *r*(47) = .68, *p* < .001, Negative world, *r*(47) = .53 *p* < .001, Self-blame, *r*(47) = .54, *p* < .001; British PTCI, *r*(48) = .65, *p* < .001, Negative self, *r*(48) = .69, *p* < .001, Negative world, *r*(48) = .43 *p* < .01, Self-blame, *r*(48) = .41, *p* < .01.

### PCSAM

#### Principal component analyses and item retention

In order to ensure that all questions on the PCSAM were measuring the same scale, we evaluated the degree to which scores on each question correlated with scores on all other questions. For an item to be retained at this stage, it had to correlate greater than *r* = .30 with at least two other items. The only item that did not meet this criterion was item number 1. Hence, this item was removed. We then submitted the other 20 items to a principal-component analysis with oblim rotation. The Kaiser-Meyer-Olkin measure verified the sampling adequacy for the analysis, KMO = .88 (Hutcheson and Sofroniou 1999), and all KMO values for individual items were > .79 and thus were well above the acceptable level (Field 2009). Bartlett’s test of sphericity χ^2^ (190) = 1462.52, *p* < .001, indicated that correlations between items was sufficiently large for principal components analysis (Field 2009). An initial analysis was conducted to obtain eigenvalues for each component in the data. Four components had eigenvalues over Kaiser’s criterion of 1 and in combination explained 71.55% of the variance^b^. However, items 8, 13, 14 and 18 did not load above .40 on any of the factors and items 2 and 20 loaded equally onto two factors. Therefore, these items were removed.

We then submitted the 14-item PCSAM to a principal-component analysis with oblim rotation. The Kaiser-Meyer-Olkin measure verified the sampling adequacy for the analysis, KMO = .85 (Hutcheson and Sofroniou 1999), and all KMO values for individual items were > .75. Bartlett’s test of sphericity χ^2^ (91) = 912.50, *p* < .001, indicated that correlations between items was sufficiently large for principal components analysis. Three components had eigenvalues over Kaiser’s criterion of 1 and in combination explained 70.86% of the variance. The first factor explained 49.96% of the variance and the second and third factors, an additional 11.71% and 9.19%, respectively. Visual examination of the scree plot also suggested a three-factor solution. Given the convergence of the scree plot and Kaiser’s criterion on three components, this was the number of components that were retained in the final analysis. Table 6 shows the factor loadings. The items that cluster on the same components suggest that component 1 represents challenge to beliefs and belonging (5 items), component 2 represents communal aspects of self (5 items) and component 3 represents public roles and identity (4 items). Thus, the final inventory contained 14-items and the components were similar to that derived from the qualitative study. The three PCSAM scales correlated moderately to strongly with each other (all *p*s < .001); Beliefs and Belonging and Communal, *r*(93) = .49, Beliefs and Belonging and Public, *r*(93) = .60, Communal and Public, *r*(93) = .56. The correlations with the Total Score were *r*(93) = .80, .85, .85, for Beliefs and Belonging, Communal, and Public, respectively.

**Table 6:** **Pattern matrix for public and communal self appraisal measure for total sample/British/Asian**

	Factor 1	Factor 2	Factor 3
Since the event I feel let down by fate/my beliefs/God/ my faith	.92/.33/.93	-.04/.10/.01	-.004/.78/-.02
My faith/religion/beliefs have been challenged by the event	.86/-.13/.92	-.07/.18/-.15	.06/.90/-.01
Since the event I feel let down by the world	.79/.64/.73	.17/-.11/.18	.04/.37/-.04
Since the event I feel I do not have a place in the world	.75/.83/.81	-.01/-.19/.003	-.20/.18/.15
Since the event I no longer feel close to others	.62/.90/.82	.08/-.04/.11	-.30/.03/.05
Since the event people have become a priority	-.12/-.10/.07	.86/.91/.70	.11/-.01/-.41
I do not want anyone to know about the event	-.02/.20/-.13	.68/.51/.74	-.08/-.04/.21
Since the event I have sacrificed my needs for the needs of significant others	.30/.15/.11	.66/.52/.74	.08/.32/-.05
Since the event my values have changed	.08/.12/.07	.60/.73/.64	-.33/.16/.47
Since the event my relationships have been damaged or challenged	.11/.55/.15	.54/.41/.70	-.33/.05/.10
Since the event I have failed in my roles	-.001/.71/.13	.01/.34/-.01	-.92/-.06/.87
Since the event I have lost my social role/identity	-.05/.73/.02	.09/.30/.15	-.90/-.02/.85
Since the event I have not lived up to social or cultural expectations	.01/.74/.05	-.01/.19/-.01	-.87/.02/.83
Since the event I do not feel I am a significant member of my culture/society/community/group	.21/.97/.44	-.06/04/-.07	-.78/-.15/.56

#### Internal consistency

Cronbach’s alphas for the three PCSAM scales and total score were as follows; total score α = .92; Beliefs and Belonging α = .90, Communal α = .81, and Public α = .92^c^.

#### Test-retest reliability

Pearson correlations were calculated to examine temporal stability of the PCSAM. The test-retest reliability was found to be excellent overall, *r*(68) = .89, *p* < .001 and for each subscale; Beliefs and Belonging, *r*(68) = .85, *p* < .001; Communal, *r*(68) = .87, *p* < .001; and Public, *r*(68) = .85, *p* < .001^d^.

#### Convergent validity

To examine the convergent validity of the PCSAM we examined correlations between PCSAM scores and the PTCI. There were significant correlations between the PCSAM and PTCI (Table 7). To examine the relationships between cognitions and posttraumatic symptoms, Pearson correlations were conducted between the PCSAM and PCL. Table 7 shows that the PCSAM was found to significantly correlate with PTSD symptoms.

**Table 7:** **Pearson correlation coefficients between PCSAM, PTCI and PCL for total sample/British/Asian**

	PCSAM			
	Beliefs	Communal	Public	Total
PTCI				
Self	.74**/.76**/.69**	.61**/.70**/.55**	.73**/.87**/.61**	.82**/.88**/.77**
World	.61**/.70**/.50**	.52**/.46**/.57**	.49**/.52**/.44**	.63**/.61**/.63**
Self-Blame	.37**/.53**/.20	.42**/.47**/.36*	.50**/.61**/.37**	.52**/.61**/.40**
Total	.74**/.80**/.65**	.64**/.67**/.60**	.72**/.82**/.61**	.82**/.86**/.78**
PCL	.52**/.49**/.50**	.53**/.60**/.48*	.72**/.79**/.70**	.71**/.73**/.70**

*Note.* *p < .05. **p < .01; PTCI = Posttraumatic Cognitions Inventory; PCSAM = Public and Communal Self Appraisal Measure; PCL = Posttraumatic Stress Disorder Checklist.

#### Discriminative validity: differences between groups

To examine whether the PCSAM could discriminate between those with and without PTSD a 2 (Culture; Asian vs. British) x 2 (PTSD status; PTSD vs. no PTSD) ANOVA was used with PSCAM total as the dependent variable. For the total score, the PTSD main effect was significant, *F*(1, 91) = 79.91, *p* < .001, ƞ_p_^2^ = .47; those with PTSD scored higher on the PCSAM than those without PTSD. The culture main effect and interaction were not significant.

2 (Culture; Asian vs. British) x 2 (PTSD status; PTSD vs. no PTSD) ANOVAs were used with PCSAM subscales as the dependent variables. For the Beliefs and Belonging subscale, the PTSD main effect was significant, *F*(1, 91) = 33.24, *p* < .001, ƞ_p_^2^ = .27; those with PTSD scored significantly higher than those without PTSD. The culture main effect was significant, *F*(1, 91) = 6.69, *p* = .01, ƞ_p_^2^ = .07; the Asian group scored significantly higher than the British group. The interaction was not significant. For the Communal subscale, the PTSD main effect was significant, *F*(1, 91) = 35.92, *p* < .001, ƞ_p_^2^ = .28; those with PTSD scored significantly higher than those without PTSD. The culture main effect and interaction were not significant. For the Public subscale, there was a significant interaction between PTSD and culture, *F*(1, 91) = 4.88, *p* = .03, ƞ_p_^2^ = .05. Post-hoc follow-up comparisons found that the British PSTD, *t*(46) = 8.36, *p* < .001, *d* = 2.24, and the Asian PTSD groups, *t*(45) = 4.29, *p* < .001, *d* = 1.25, scored significantly higher than their non-PTSD comparison groups. It was found that the Asian PSTD and British PTSD groups did not significantly differ. However, the Asian no PTSD group scored significantly higher than the British no PTSD group, *t*(59) = 2.01, *p* < .05, *d* = .51.

Lastly, a discriminant function analysis was conducted to examine the specificity and sensitivity of the PSCAM subscales in identifying individuals with and without PTSD. The three obtained PSCAM factors loaded on one function which classified 80% of the sample correctly into those with and without PTSD, Wilks’ λ = .53, χ^2^ (3, *N* = 95) = 58.55, *p* < .001. Sensitivity was .77 and specificity was .81. The discriminant function analyses were also conducted for the British and Asian groups separately. For the British group, the three obtained PSCAM factors loaded on one function which classified 83% of the sample correctly into those with and without PTSD, Wilks’ λ = .54, χ^2^ (3, *N* = 48) = 28.35, *p* < .001. Sensitivity was .73 and specificity was .88. For the Asian group, the three obtained PSCAM factors loaded on one function which classified 79% of the sample correctly into those with and without PTSD, Wilks’ λ = .53, χ^2^ (3, *N* = 47) = 28.54, *p* < .001. Sensitivity was .74 and specificity was .82.

#### Stability of the factor structure of the PCSAM for Asian and British participants

The factor structure of the 14-item PCSAM was tested in Asian and British trauma survivors. Given the small sample sizes these analyses were exploratory. As seen in Table 6, for the Asian group all items had high loadings on the factor to which they had been assigned. Very few showed substantial correlations on other factors. When the analysis was conducted for the British sample a different pattern of loading emerged. While three factors still emerged (which explained 72.86% of the variance; first factor explained 54.49% of the variance and the second and third factors, an additional 10.38% and 7.99%, respectively) for the British group, the items clustered quite differently. The items that clustered on the same components suggest that component 1 represented belongingness (the items relating to public self loaded onto this factor), component 2 still represented communal aspects of self and component 3 represented faith/spiritual beliefs. Hence, the components seemed to be culturally influenced and the PCSAM sub-scales described above may be more appropriate for use in Asian populations^e^.

## Discussion

Study 2 again found that the cultural influences on appraisals tended to extend to trauma memories. Specifically, Asian trauma survivors reported higher levels of pleasantness and legitimacy appraisals and lower levels of anticipated effort, goal/need conduciveness and attentional activity than the British group. This supports previous research that suggests Western cultures generally emphasize appraisals of anticipated effort and Asian cultures tend to appraise situations to be more legitimate when compared to Western cultures (Mauro et al. 1992; Mesquita and Walker 2003). Given the role of appraisals in PTSD, Study 2 was also interested in appraisals that differentiated between those with and without PTSD. Those with and without PTSD did not differ in their appraisals of attentional activity, certainty and coping associated with the negative memory. However, for the trauma memory those with PTSD reported fewer appraisals of attentional activity, certainty and coping than those without PTSD. Furthermore, those without PTSD appraised less personal responsibility for the trauma event. These differences between those with and without PTSD were evident regardless of one’s cultural background suggesting cultural similarities in the dysfunction appraisals of those with PTSD. The only appraisal type that differed cross-culturally was control; appraisals of control only differentiated between British trauma survivors with and without PTSD for the trauma memory. This aligns with Western cultures valuing control and violations of cultural expectations resulting in psychological distress (e.g. Jobson and O’Kearney 2009; Mesquita and Walker 2003).

Second, we examined trauma-specific appraisals. We found that, unlike Study 1, those with PTSD, regardless of cultural background, scored significantly higher on the PTCI than those without PTSD. Therefore, the PTCI seems appropriate for use with Asian trauma survivors with PTSD. Those with PTSD may hold culturally similar dysfunctional negative appraisals about the self, world and self-blame. Third, we investigated the usefulness of a new measure developed to investigate trauma-associated appraisals in terms of more public and communal aspects of self. The 14-item questionnaire loaded onto three factors (challenges to beliefs and belonging, communal, public and social roles). Internal consistency, convergent validity and test-retest reliability were good. The PCSAM was able to discriminate between those with and without PTSD.

## General discussion

The aim of this research was to investigate the influence of culture on cognitive appraisals associated with trauma experiences and the influence of these appraisals on posttraumatic psychological adjustment in a non-clinical sample (Study 1) and in a sample of trauma survivors with and without PTSD (Study 2). The findings firstly demonstrated cultural differences in the way experiences are appraised. British participants were found to appraise significantly less pleasantness (Study 1 and 2) and legitimacy (Study 2) and significantly greater anticipated effort (Study 1), goal-need conduciveness (Study 2), norm-self compatibility (Study 1), and attentional activity (Study 1) than Asian participants. These differences reflect what has been found in previous research and demonstrate that such differences extend to the trauma memory. Such cultural differences reflect British participants valuing agency, independence, assuming their reactions are typical and being less concerned about discrepancies with the reactions of others (Markus and Kitayama 2010; Mauro et al. 1992; Mesquita and Walker 2003). In both studies Asian participants rated events as more pleasant, which could potentially be explained by a culture-based appraisal bias. Mesquita and Fridja (1992) assert that this cultural difference may occur due to individuals from different cultures evaluating the event differently; particular appraisals are assigned more importance in one culture than another. Alternatively, the nature of the appraisal may differ across cultures, whereby individuals may evaluate an event similarly, however, while both may appraise the event to be unpleasant, unpleasantness may be more unpleasant for one person than for another (Mauro et al. 1992; Schimmack et al. 2002). Additionally, Asian cultures tend to have greater acceptance of situation outcomes and fate (Mesquita and Walker 2003). Therefore, appraisals, including those associated with trauma experiences, are in line with what is culturally emphasized and expected and thus, appear to function to develop, express and maintain the culturally-expected self (Mesquita and Walker 2003).

Despite these cultural differences in appraisals, the findings overall also suggest that the relationships between cognitive appraisals and PTSD symptoms are predominately culturally similar. Those with and without PTSD, regardless of their cultural background, were found to appraise events differently; those with PTSD appraised their memories to be less pleasant with greater anticipated effort than those without PTSD. Additionally, those with PTSD were found to appraise the trauma memory uniquely. While those with and without PTSD tended to appraise the negative event similarly, those with PTSD appraised that they could not cope as well in the trauma event, perceived the trauma event to be less predictable, certain and understandable, and appraised that they had less motivation to attend closely to the event than trauma survivors who did not develop PTSD. Furthermore, those with PTSD felt they were personally responsible for the trauma event.

Substantial research has demonstrated the role of control appraisals in maintaining PTSD. However, cross-cultural research has demonstrated that control is one particular cognitive appraisal that is valued to a greater extent in Western cultures than Asian cultures (e.g. Mesquita and Walker 2003). This cultural difference was found to influence the relationship between control and PTSD. In Study 1 while a significant negative correlation was found between lower levels of perceived control and PTSD symptoms, this was only found to be the case for the British group. In Study 2, while British trauma survivors with and without PTSD did not differ significantly in their appraisals of control associated with the negative memory, for the trauma memory British trauma survivors with PTSD reported lower levels of control appraisals in the trauma memory than those without PTSD. In contrast, Asian trauma survivors with and without PTSD did not differ significantly for either the negative or trauma memories. Thus, as perceived personal control and agency are valued in Western cultures, appraisals associated with situations, such as the trauma event, that violate culturally expected cognitive appraisals are potentially distressing (Mesquita and Walker 2003). Therefore, perceived control differentiates between those with and without PTSD in British cultures but not Asian cultures.

In terms of trauma-specific appraisals, Study 1 found that for the British group, the PTCI was significantly correlated, and predicted, PTSD symptoms. In contrast, for the Asian group the PTCI did not significantly predict PTSD symptoms. Based on this finding and the findings of a related qualitative study, it was proposed that the PTCI may be tapping into individualistic type appraisals (e.g. I am a weak person, I can’t rely on myself, I am inadequate) rather than interdependent, public (i.e. social roles) and communal (relationships and interdependence) appraisals which are emphasised in Asian cultures. However, in Study 2 the PTCI was found to differentiate between those with and without PTSD, regardless of cultural background. Therefore, in clinical samples the PTCI may be appropriate for use with Asian trauma survivors as those with PTSD may hold culturally similar dysfunctional negative appraisals about the self, world and self-blame.

The final aim of Study 2 was to investigate the reliability and validity of the PCSAM and its appropriateness for use in Asian trauma survivor populations. The PCSAM was a newly developed measure aimed to assess the influence of trauma on more public and communal aspects of self. The final PCSAM inventory consisted of 14-items that loaded onto three components; 1) beliefs and belonging, 2) communal aspects of self, and 3) public and social roles. The PSCAM was found to have good internal consistency, test-retest reliability and convergent validity. In regards to discriminate validity, the PCSAM (and its sub-scales) could discriminate between those with and without PTSD. A discriminant function analysis found that the specificity and sensitivity of the PSCAM subscales in identifying individuals with and without PTSD was good. Finally, while the sample sizes were small, and hence the analyses were exploratory, the factor structure of the 14-item PCSAM was explored in Asian and British trauma survivors. For the Asian group all items had high loadings on the factor to which they had been assigned. When the analysis was conducted for the British sample a different pattern of loading emerged; most of the items relating to public self loaded onto the belonging factor in the British group suggesting that public aspects of self may be conceptualized culturally differently in Asian and British participants and hence, the PCSAM sub-scales may be more appropriate for use in Asian samples. Further research is required to explore this.

### Theoretical and clinical implications

This study supports PTSD models emphasis on the role of cognitive appraisals in PTSD (Ehlers and Clark 2000) and the focus on appraisals in the treatment of PTSD (Resick 2001). The results extend findings conducted with Western populations to indicate that appraisals also play an important role in PTSD in Asian cultures, which is expected given the emphasis theories of emotion give to the role of cognition in emotion (e.g. see Mauro et al. 1992). The results indicate that the cultural differences in cognitive appraisals of everyday events, which are in line with cross-cultural theories (Mesquita and Walker 2003), tend to extend to trauma cognitive appraisals. Nonetheless, despite these cultural differences in trauma cognitive appraisals, the findings suggest that the types of cognitive appraisals that relate to PTSD symptoms may be culturally similar. However, appraisals of personal control seemed to be somewhat unique. While appraisals of personal control were found, as in previous research (e.g. Jobson and O’Kearney 2009), to differentiate between British trauma survivors with and without PTSD, appraisals of control had little relevance in discriminating between Asian trauma survivors with and without PTSD. This suggests that in some instances the influence of cultural differences in self-construal on cognitive appraisals influences PTSD outcome (see Jobson 2009). The success of the PCSAM in differentiating between those with and without PTSD demonstrates the importance of also considering also public and communal aspects of self in those with PTSD.

Effective treatment for PTSD targets appraisals of trauma (Resick 2001). The effectiveness of these interventions has been demonstrated in Western cultures (e.g. Basoglu et al. 2007; Duffy et al. 2007). However our understanding of interventions for non-Western groups is exceptionally limited. Therefore, research improving our understanding of the processes involved in PTSD for those from different cultural groups is imperative for generalizing current interventions. Given the focus of effective treatments on appraisals, it is important that clinical practice and research consider the cross-cultural research highlighting the influence of culture on appraisals and associated emotional responses. The findings suggesting many appraisals associated with PTSD are culturally similar indicate that many of the treatment targets may be generalizable. However, it remains important that clinicians consider how trauma appraisals may challenge cultural norms and culturally influenced self (including self in relation to others) of a client. Thus, cognitive restructuring in therapy may need to focus on realigning sufferers’ beliefs with their culturally-determined conceptual self. It may be important to include more social role, group and interpersonal appraisals (and less focus on control) as potential moderators of PTSD within Asian cultures and thus, target these appraisals in treatment. Furthermore, current measures assessing trauma-related appraisals may benefit from including greater focus on appraisals associated with interdependence. Finally, the recent changes to the PTSD criteria in DSM-V (American Psychiatric Association 2013) includes negative alterations in cognitions and persistent and distorted blame of self or others which seems to be appropriate cross-culturally as those with PTSD, regardless of cultural background, had negative cognitions about self (private, public and communal), world, and self-blame.

## Limitations and conclusions

We acknowledge there are several limitations to these studies. First, participants completing the task in English may have influenced findings. However in both studies groups were equivalent in how difficult they found tasks. Additionally, the groups may have differed on demographic features other than culture. For instance, in Study 2 the groups differed in terms of age and gender, which may have influenced findings. However, when we conducted the analyses taking into account these differences a similar pattern of results emerged. Second, the cross-sectional designs prevented causal inferences being developed. As retrospective studies, the time since the recalled events could have been a confounding variable. Third, future research would benefit from investigating the influence of culture on appraisals associated with particular traumas as trauma type (e.g. interpersonal) may have had an influence on findings. Fourth, it was difficult to estimate selection bias. Fifth, this research, as in most other cross-cultural research (Mesquita and Walker 2003), focuses on attribution to a specific agent (self or other). Agency and agency appraisals can also be associated with magic spells, spirits, fate, and so forth. Such appraisals need further exploration in relation to culture and appraisals of trauma. This especially needs to be considered in terms of cultural differences in religious beliefs. A significant factor regarding the samples was group heterogeneity (i.e. the Asian group was comprised of several cultural groups). This is keeping with previous studies (Hall et al. 2004; Jobson and O’Kearney 2009; Wang and Ross 2005) and while, this approach was selected, as this is the first study to explore these issues, the next step is to use more homogeneous groups and to include a measure of individualism-collectivism. Furthermore, future studies should use larger sample sizes, especially in regards to the preliminary principal component analyses (PCA) used in Study 2. PCA was used due to the study using a relatively small sample size compared to what is advised for exploratory factor analyses. Further, in order to explore the data without any preconceived theories as to the relationships between variables, it was deemed PCA would be most appropriate as it allows for the inclusion of all variance. PCA thereby enabled the exploration of what patterns emerged in the data to reveal the internal structure of the data and to find what patterns emerge in all of the variance, including variance unique to each variable, variance common among variables, and error variance, which exploratory factor analyses would not have allowed (Tabachnick and Fidell 2007). However, going forward with a larger sample and the final 14-item PCSAM, exploratory factor analysis would be advantageous to analyse the covariance and factor structure of the PCSAM. Finally, all participants were residing in the UK, and had lived in the UK for varying times, which may influence findings. Future studies should investigate these issues using participants in their country of origin. Despite these limitations these studies, as far as we are aware, are some of the first to investigate the role of culture in trauma appraisals and associated posttraumatic psychological adjustment. The findings suggest that while there are cultural differences in appraisals of trauma experiences, those with PTSD, regardless of cultural background, may have similar dysfunctional appraisals, which may play a role in the development and maintenance of PTSD. This is initial research in this area and thus, further research is required to further investigate this important area.

### Consent

Written informed consent was obtained from the participants for the publication of this report and any accompanying images.

## Endnotes

^a^Contact first author for further details about the qualitative study.

^b^The first factor explained 50.54% of the variance and the second, third and fourth factors, an additional 8.78%, 7.15%, and 5.08%, respectively.

^c^We also examined Cronbach’s alphas for the Asian and British groups separately; Asian - total α = .90; Beliefs and Belonging α = .91, Communal α = .79, Public α = .90; British - total α = .93; Beliefs and Belonging α = .86, Communal α = .82, Public α = .93.

^d^For each cultural group, test-retest reliability was found to be excellent overall, British *r*(34) = .94, *p* < .001; Asian *r*(32) = .83, *p* < .001, and for each subscale; Beliefs and Belonging, British *r*(35) = .81, *p* < .001; Asian *r*(32) = .84, *p* < .001; Communal, British *r*(35) = .90, *p* < .001; Asian *r*(32) = .82, *p* < .001; and Public, British *r*(35) = .95, *p* < .001; Asian *r*(32) = .78, *p* < .001.

^e^Cronbach’s alphas for Belonging and Spiritual for the British group; Spiritual α = .80, Belonging α = .95. Test-retest reliability Spiritual, *r*(37) = .71, *p* < .001, and Belonging, *r*(37) = .91; *p* < .001.
